# Effects of a robot‐aided somatosensory training on proprioception and motor function in stroke survivors

**DOI:** 10.1186/s12984-021-00871-x

**Published:** 2021-05-10

**Authors:** I-Ling Yeh, Jessica Holst-Wolf, Naveen Elangovan, Anna Vera Cuppone, Kamakshi Lakshminarayan, Leonardo Capello, Lorenzo Masia, Jürgen Konczak

**Affiliations:** 1 Health and Social Sciences Cluster, Singapore Institute of Technology, Singapore, Singapore; 2 Human Sensorimotor Control Laboratory, School of Kinesiology, University of Minnesota, Minneapolis, USA; 3 Department of Robotics, Brain and Cognitive Sciences, Istituto Italiano di Tecnologia, Genova, Italy; 4 Department of Neurology and School of Public Health, University of Minnesota, Minneapolis, USA; 5 The BioRobotics Institute, Scuola Superiore Sant’Anna, Pisa, Italy; 6 Department of Excellence in Robotics and AI, Scuola Superiore Sant’Anna, Pisa, Italy; 7 Institut für Technische Informatik, Universität Heidelberg, Heidelberg, Germany

**Keywords:** Human, Somatosensation, Upper limb, Rehabilitation, Cerebrovascular disease/stroke

## Abstract

**Background:**

Proprioceptive deficits after stroke are associated with poor upper limb function, slower motor recovery, and decreased self-care ability. Improving proprioception should enhance motor control in stroke survivors, but current evidence is inconclusive. Thus, this study examined whether a robot-aided somatosensory-based training requiring increasingly accurate active wrist movements improves proprioceptive acuity as well as motor performance in chronic stroke.

**Methods:**

Twelve adults with chronic stroke completed a 2-day training (age range: 42–74 years; median time-after-stroke: 12 months; median Fugl–Meyer UE: 65). Retention was assessed at Day 5. Grasping the handle of a wrist-robotic exoskeleton, participants trained to roll a virtual ball to a target through continuous wrist adduction/abduction movements. During training vision was occluded, but participants received real-time, vibro-tactile feedback on their forearm about ball position and speed. Primary outcome was the just-noticeable-difference (JND) wrist position sense threshold as a measure of proprioceptive acuity. Secondary outcomes were spatial error in an untrained wrist tracing task and somatosensory-evoked potentials (SEP) as a neural correlate of proprioceptive function. Ten neurologically-intact adults were recruited to serve as non-stroke controls for matched age, gender and hand dominance (age range: 44 to 79 years; 6 women, 4 men).

**Results:**

Participants significantly reduced JND thresholds at posttest and retention (Stroke group: pretest: mean: 1.77° [SD: 0.54°] to posttest mean: 1.38° [0.34°]; Control group: 1.50° [0.46°] to posttest mean: 1.45° [SD: 0.54°]; F[2,37] = 4.54, *p* = 0.017, η_p_^2^ = 0.20) in both groups. A higher pretest JND threshold was associated with a higher threshold reduction at posttest and retention (*r* = − 0.86, − 0.90, *p* ≤ 0.001) among the stroke participants. Error in the untrained tracing task was reduced by 22 % at posttest, yielding an effect size of *w* = 0.13. Stroke participants exhibited significantly reduced P27-N30 peak-to-peak SEP amplitude at pretest (*U* = 11, *p* = 0.03) compared to the non-stroke group. SEP measures did not change systematically with training.

**Conclusions:**

This study provides proof-of-concept that non-visual, proprioceptive training can induce fast, measurable improvements in proprioceptive function in chronic stroke survivors. There is encouraging but inconclusive evidence that such somatosensory learning transfers to untrained motor tasks.

*Trial registration* Clinicaltrials.gov; Registration ID: NCT02565407; Date of registration: 01/10/2015; URL: https://clinicaltrials.gov/ct2/show/NCT02565407.

## Background

Nearly two-thirds of stroke survivors exhibit forms of somatosensory or proprioceptive dysfunction [1, 2]. Proprioceptive deficits are related to longer length-of-stay in hospitals [3], poor quality of movement, poorer activities of daily (ADL) function and reduced participation in physical activity [4–6]. Proprioceptive deficits predict treatment responses to robot-assisted motor retraining with augmented proprioceptive feedback [7] These may be explained by the crucial role of proprioception in motor control and learning [8, 9]. Proprioceptive training is a form of somatosensory intervention that aims to enhance proprioceptive function. Several forms of somatosensory intervention such as passive, repetitive cutaneous stimulation [10, 11], passive limb movement training [12], repeated somatosensory discrimination practice and active sensorimotor training with augmented somatosensory feedback [7, 13, 14, 15] have been proposed to aid recovery of proprioceptive function and motor function after stroke. Proprioceptive improvements observed after proprioceptive training interventions correlated with improvement of untrained motor performance in healthy young adults [16, 17]. This further supports the rationale to implement proprioceptive-motor training for people with stroke. Among all types of proprioceptive intervention, active sensorimotor training with augmented somatosensory feedback [7, 13–15] seem to produce consistent results across studies [1, 18]. These interventions often employ somatosensory signals either to replace visual feedback on motor performance or to augment existing visual and somatosensory feedback for online motor control. One well studied mode of somatosensory feedback is vibro-tactile feedback (VTF) applied to the skin surface. Incorporating VTF with movement training has been shown to improve the learning of simple motor tasks in healthy adults and clinical populations [19–21]. There is evidence that it can effectively enhance proprioceptive function [22].

Somatosensory evoked potentials (SEPs) recorded via electroencephalography (EEG) are an objective neurophysiological marker of somatosensory processing with established procedures and normative values that has been used among clinical populations. Adults after stroke typically present with a lower peak amplitude or longer peak latency of SEPs (e.g. [23, 24]). Moreover, the restoration of typical SEPs has been reported following somatosensory interventions [25, 26]. Thus, we here recorded SEP to verify changes in the neural processing of somatosensory signals in sensorimotor cortex as a function of the somatosensory-motor intervention employed in this project

**Fig. 1:**
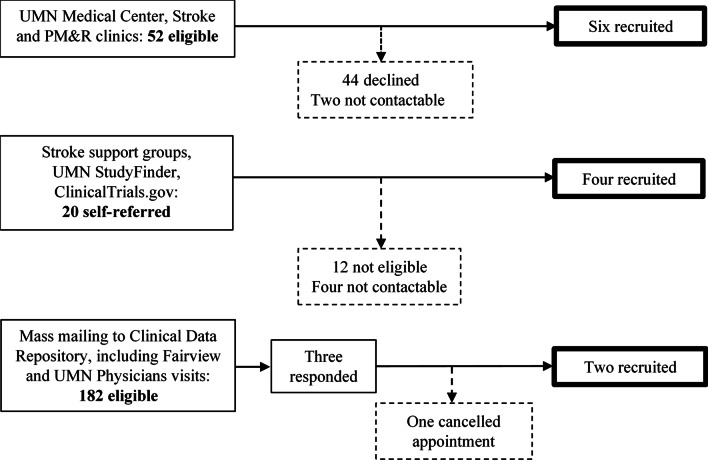
Recruitment flowchart. *UMN* University of Minnesota, *PM&R* Physical medicine and rehabilitation

.

To address if a somatosensory-based training is a meaningful approach for the rehabilitation of proprioceptive and motor function after stroke, this proof-of-concept study aimed to determine whether wrist proprioception could be trained in stroke survivors and whether such sensory learning transfers to other functional tasks involving the same joint and limb motor system.

## Methods

### Participants

Twelve stroke survivors at least three months post cerebral stroke were recruited (Table 1). Study inclusion required an ability to achieve 20° active movement of wrist ab/adduction at the more-affected side in the gravity-eliminated position (a score of 2 or greater out of 5 in manual muscle testing) [27]. Exclusion criteria were: (1) non-cerebral stroke, (2) < 23 points on Mini Mental State Examination [28], (3) markedly increased muscle tone as indicated by > 1 + on the Modified Ashworth Scale [29], (4) other medical conditions affecting upper limb sensorimotor function, (5) inability to perceive VTF consistently on either forearm, and (6) lack of MRI records confirming stroke diagnosis. Participants were not screened for proprioceptive dysfunction using existing clinical measures as existing clinical measures are not be sensitive enough to detect proprioceptive impairment. Participants were recruited via an outpatient neurology clinic, local stroke support groups, and mailing to addresses retrieved from the clinical data depository of the University of Minnesota Clinical and Translational Science Institute (Fig. 1). All participants lived at home and were independent in self-care. Ten neurologically-intact adults matched by age, gender, and hand dominance were recruited to serve as non-stroke controls for age and gender (median age: 71 years, range: 44 to 79 years; 6 women, 4 men). The study protocol was approved by the Human Research Protection Program of University of Minnesota. Written informed consent was obtained from all participants prior to data collection. Study data are available from the corresponding author upon reasonable request.

**Fig. 2:**
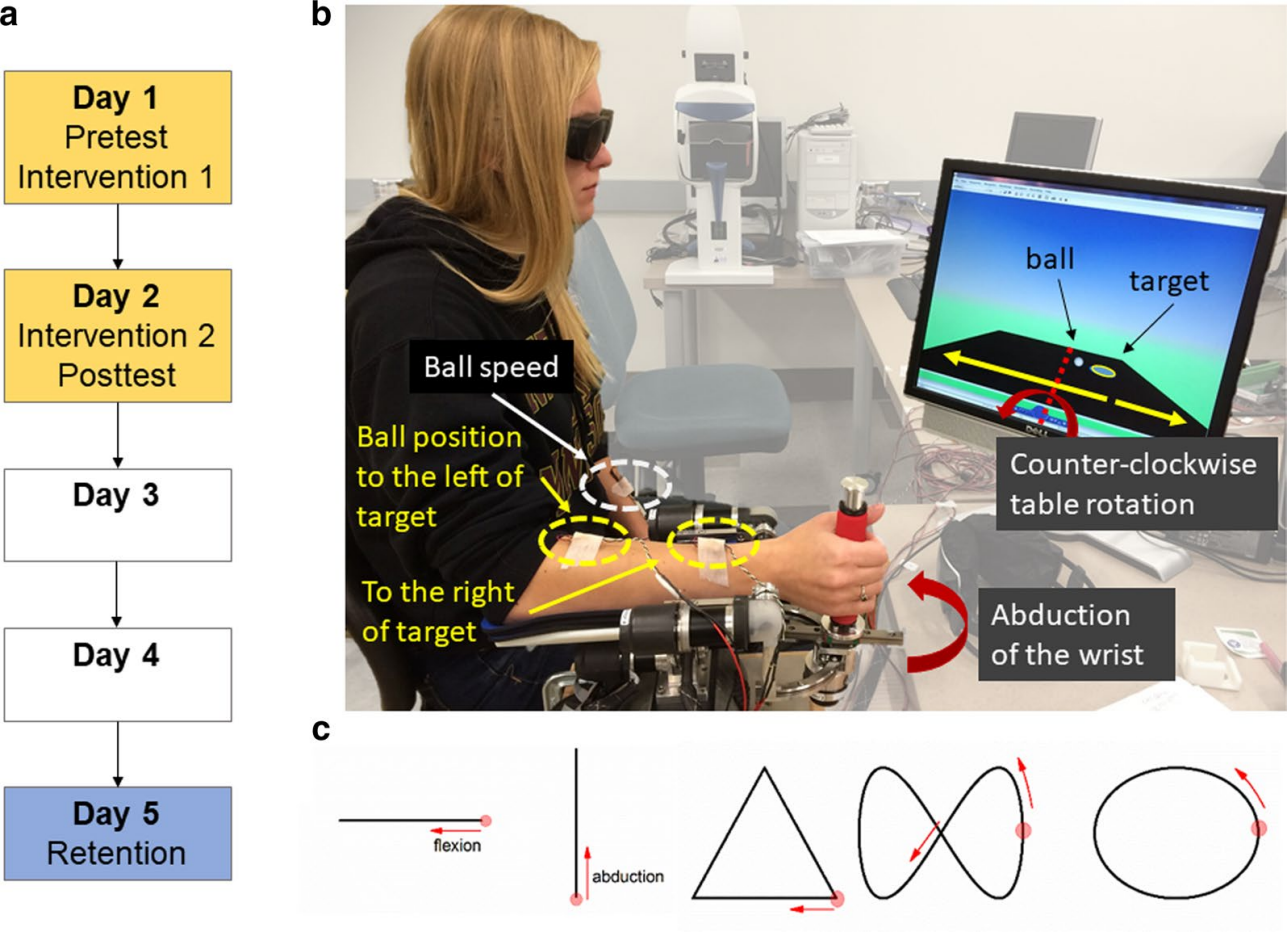
**a** Study timeline. **b** Experimental setup of the robot and the virtual ball balancing task. Wrist abduction tilted the virtual table seen in the computer display toward the left, adduction toward the right, as indicated by the maroon arrows. The task was to move the virtual ball rolled into the blue target circle. The two vibration motors attached to the skin arm indicated the respective ball position relative to the target. The distal motor turned on when the ball was on the right side of the target, the proximal motor when the ball was to the left side. Distance from the target was frequency coded (frequency increased with increasing distance to the target. The motor on the non-trained side indicated the ball speed. **c** Figures of the untrained wrist tracing task. Red circles and arrows indicate the starting point and movement direction for right-handed users

**Table 1:** Demographics and clinical evaluation for participants with stroke

ID	Gender	Age (years)	Time post stroke (months)	Lesion side	Lesion location	Type	FMA-UL (0–66)
S03	W	57	27	L	Cortical and subcortical parietal lobe	Ischemic	66
S04	M	73	11	L	EC, putamen, PVWM	Ischemic	66
S05	M	47	4	L	Posterior subcortical frontal, BG, posterior limb of IC	Ischemic	65
S06	W	74	6	R	Thalamus, putamen	Hemorrhagic	64
S07	M	63	7	L	Corona radiate	Ischemic	65
S08	W	42	13	R	Superior thalamus, cortical and subcortical temporal and occipital lobe	Ischemic	64
S09	W	63	5	R	Frontal (precentral gyrus), parietal (postcentral gyrus), occipital lobe	Ischemic	66
S10	M	65	26	L & R	Cortical and subcortical occipital lobe, L & R thalamus	Ischemic	46*
S11	M	71	55	R	Thalamus	Hemorrhagic	42*
S12	W	68	6	L	Frontal (precentral gyrus)	Ischemic	65
S13	M	60	49	L	Subcortical frontal and parietal	Ischemic	58
S14	W	56	14	L	Frontal (precentral gyrus), parietal (postcentral gyrus)	Ischemic	64
Ave.	6 Women/6 men	62	18	4 R/ 7 L / 1 both	3 Cortical / 7 subcortical/ 2 both	10 Ischemic	61

*FMA-UL* Fugl-Meyer Assessment Upper Limb, *EC* external capsule, *PVWM* periventricular white matter, *BG* basal ganglia, *IC* internal capsule. *Impaired wrist position sense indicated by Erasmus MC modified Nottingham Sensory Assessment

### Study design

The study employed a pre-post design with a single control group. Participants completed the pretest and one intervention session on Day 1, the second intervention and the posttest on Day 2 with retention testing at Day 5 (Fig. 2a).

**Fig. 3:**
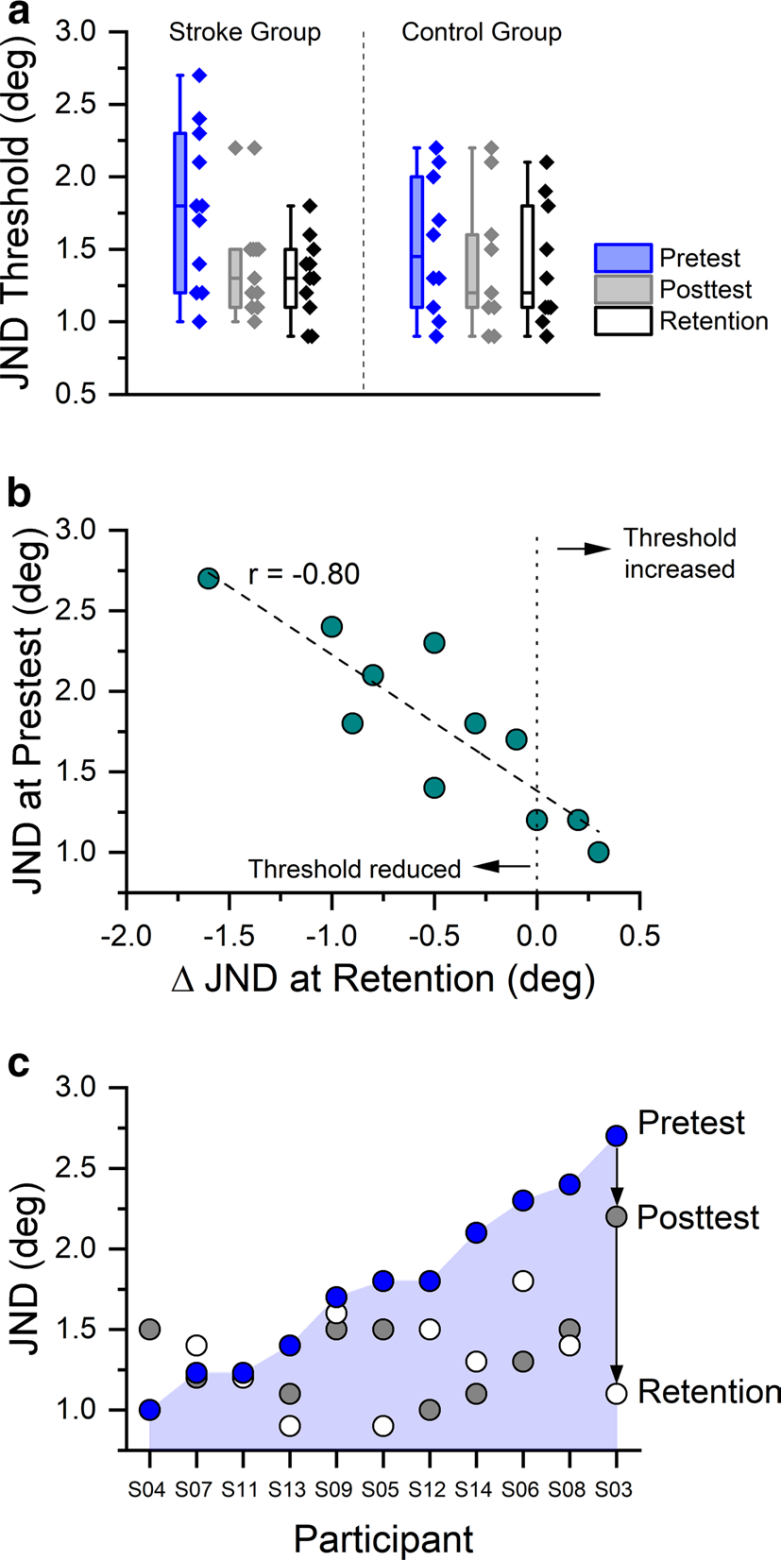
**a** Boxplot of Just-Noticeable Difference (JND) position sense thresholds at pretest, posttest and retention for both groups. Each box indicates the interquartile range (IQR). The line within the box indicates the median. Whiskers represent the 1st and 99th percentile. Adjacent diamond symbols show all individual subject JNDs. **b** Correlation between JND thresholds at pretest in relation to change in JND at retention. A negative value indicates a reduction in threshold, i.e. an improvement in proprioceptive acuity. **c** Change in JND for each participant as a function of training. Data are sorted in ascending order for the pretest value. Grey and white circles indicate the related values at posttest and retention

### Apparatus

A wrist/hand exoskeleton robotic system (the *WristBot*) allowing full, passive ROM at three degrees-of-freedom (DOF; wrist flexion/extension, wrist abduction/adduction and forearm pronation/supination; see Fig. 2b) was used for training and assessment (for a full technical description of the robot see [30, 31]). Optical encoders measured angular displacement at a high resolution (0.0065° in wrist abduction/adduction (AA); 0.0075° in wrist flexion/extension (FE) [32]. The *Wristbot* was able to generate maximum torques up to 1.53 Nm in the wrist FE direction and 1.63 Nm in the wrist AA. However, the used control paradigm was implemented for a position mode to render angular displacements of preset amplitude and speed which was presented to move the user’s wrist. Robotic control was implemented through Matlab Simulink code (MathWorks, Natick, Massachusetts, USA).

### Intervention

Participants sat on a height-adjustable chair. The medial-lateral wrist joint axis was aligned with the axis of rotation of the *WristBot*. During intervention, only one degree of freedom - AA was trained. Participants grasped the robot handle and made continuous, small amplitude wrist AA movements to position a virtual ball into a target area on a tilt-able table viewed on a display (Fig. 2b). The rotation scaling factor was set to translate each degree of wrist motion to one degree of tilt angle of the virtual table. Motion of the virtual ball towards the target was generated by tilting the table using FE and AA corresponding to the two coordinated axes of rotations: the dynamics of the virtual ball was simulated by considering its mass and gravity force generated by the inclination of the table and, consequently imposing a kinematics on its trajectory. Participants completed a single 24-minute session on Day1 and Day2 for a total of 48 min of training. Both sessions began with a 5-minute familiarization phase, then continued with three 8-minute training blocks. During the familiarization phase, participants learned to associate VTF with the ball-target distance and ball speed in the presence of visual feedback of the virtual table/ball system. After familiarization with vision, participants continued practicing with eyes covered for the remainder of training relying solely on vibro-tactile feedback to complete the task of moving the virtual ball to the target zone. VTF was provided by three light-weight vibratory motors (9 mm in diameter, 25 mm in length, 4.6 g; Model 307−100, Precision Microdrives Ltd., London, United Kingdom). Two vibratory motors were placed along the longitudinal axis of the training forearm at a distance that users verbally reported that they could differentiate (Fig. 2b). They encoded ball position and distance of the ball relative to the target. Vibration frequency increased in three levels (Level 1: 80 Hz; Level 2: 90 Hz; Level 3: trains of 100-Hz pulses) with the distance from the ball to the target. Preliminary work from our group established that a 10 Hz difference in signal is discernible on the forearm and all participants reported that they were able to differentiate the differences in vibration frequency. The distal vibrator turned on when the ball was on the right side of the target, while the proximal vibration motor was switched on when the ball was the left side of target. A third vibrator encoded ball speed by vibrating between 75 and 98 Hz (Higher frequency indicated higher ball speed). This vibrator was placed on the non-performing forearm for the ease of differentiating from the other two vibrators. A trial was completed when the ball stayed within the target area for 5 s. Target locations on the table changed between trials (left, center, right). The difficulty level increased after every 6 trials by altering the virtual dynamics of the system (i.e., increasing ball mass and speed gain, decreasing table friction).

### Primary outcome measure

#### Just-noticeable difference (JND) threshold

Participants sat in a height-adjustable chair. The tested forearm was secured with a Velcro strap to the splint of the robot to minimize movement during testing. Vision was occluded. Pink noise provided via headphones masked external sounds that could provide position information. The participant’s wrist was displaced from an initial position of 10° wrist adduction (i.e. ulnar deviation) at a constant angular velocity of 6°/s. The angular speed has been set in order to not evoke stretch reflex in the wrist joint which is passively moved by the device [33]. Two stimuli were presented in each trial: A reference position of 5° wrist abduction and a comparison position. The comparison position ranged from 5.4° to 12.6° and was always more abducted than the reference position. The order of the two positions was randomized. In each trial, participants verbally identified the stimulus with the larger amplitude in response to “Which position was the farthest from the starting position?” The stimuli difference in the subsequent trial was determined based on the participant’s response by an adaptive psi-marginal algorithm [34]. A correct response was followed by a smaller stimulus difference than the previous trial and vice versa. Trial-by-trial variability ranged from 0.01° to 1.6°. For the first trial, stimulus difference was set at 1.9°, which was 25 % higher than the threshold of a healthy young adult cohort [32]. Breaks were scheduled every 10 to 15 trials. The JND test took approximately 45 min. The JND threshold represented the smallest stimulus intensity that the participant can discriminate based on the fitted performance function after 30 trials. This robot-assisted JND threshold testing assessment was chosen, because it produces less variable outcomes compared to clinical rating scale measures [2] and simple joint position matching methods [35]. An additional advantage of JND threshold testing is that it is based on passive movement and therefore is not affected by the extent of motor impairment of a stroke participant. The method applied here has established psychometric properties: test-retest reliability was *r* = 0.99 between 1st and 2nd tests; *r* = 0.97 between 1st and 3rd tests. The average within-subject variability was 0.09° [32].

### Secondary outcome measures

#### Accuracy of wrist tracing

To examine the transfer effect of the proposed wrist proprioceptive training on the untrained motor task, participants held the handle of the device and actively traced templates presented on a computer monitor by using the *WristBot* to control a cursor on the monitor. Wrist flexion/extension was mapped to linear horizontal, wrist abduction/adduction on to linear vertical cursor movement. The task consisted of 5 shapes: horizontal line, vertical line, triangle, figure of eight, and ellipse (Fig. 2c). Shapes were scaled to 60 % of the participant’s active ROM in the respective DOF to avoid confounding by end-range muscle tightness. The reference trace template was always visible. A target circle was presented to indicate the desired tracing direction. Participants started tracing with a wrist flexion movement. The obtained angular position time-series data were filtered offline using a low-pass 4th-order Butterworth filter (cut-off frequency: 2.5 Hz). The minimal distance of each cursor sampling point with respect to the template indicated the instantaneous tracing error. For each shape, the mean and standard deviation of the instantaneous tracing errors were calculated for each participant and used as variables for subsequent analysis. While the presence of visual feedback mitigates effects of the proprioceptive impairment, people with proprioceptive impairment still demonstrate observable differences in kinematics in visuomotor tasks [9, 36]. Thus, we expected the motor performance of stroke participants to change with proprioceptive training.

#### Somatosensory evoked potentials

To obtain a neural correlate of proprioceptive function, we recorded somatosensory evoked potentials. SEPs were induced by median nerve stimulation applied to the trained wrist via electrical stimulation (S88 stimulator with SIU 5 stimulus isolation unit; Grass Technologies, West Warwick, RI, USA). Square-wave pulses of 0.2 ms duration were delivered at 2 Hz and at the voltage sufficient to induce a noticeable thumb adductor twitch. 1200 stimuli were delivered in two blocks, with a break at the 600th stimulus.

EEG data were recorded continuously from nine Ag/AgCl electrodes mounted on an elastic cap using the ANT Neuro eego system (Medical Imaging Solutions GmbH, Berlin, Germany). The montage covered the primary sensorimotor cortical area (Fz, F3/4, FC1/2, FC3/4, Cz, C3/4, CP3/4, CP5/6, P3/4) on the contralateral hemisphere of the stimulated wrist and bilateral mastoid processes based on the standard international 10–20 system. Signals recorded from bilateral mastoid processes were used to re-reference the scalp recording offline. All signals were sampled at 2 kHz or 4 kHz with a 24-bit A/D-converter. EEG data were processed using EEGLAB [37] and ERPLAB toolboxes [38]. First, continuous EEG signals were visually inspected to remove visible electromyographic or movement artifacts. Second, data were resampled to 1000 Hz and baseline correction was performed using the average value. Third, data were filtered using a 2nd-order Butterworth high-pass (cut-off: 0.1 Hz) and a low-pass (cut-off: 200 Hz) filter in series. Signals were then re-referenced to the average signals recorded from bilateral mastoid processes. Lastly, the continuous signals were segmented into 300-ms epochs with 100 ms before and 200 ms after the onset of the electrical stimulus. Artifact rejection was performed through a moving average method that flagged epochs containing peak-to-peak amplitudes higher than 100 µV in 200-ms moving window in a 100-ms step. Artifact-free epochs were then averaged to generate the grand average for each participant session (89 % of total epochs were accepted after artifact rejection).

The following three measures were extracted from SEP waveforms for each participant as markers of somatosensory cortical processing: (1) peak latency of N30, defined as the first negative peak from the frontal electrodes (F3/4, FC1/2, FC3/4) after 28 ms [39], (2) peak-to-peak amplitude of P27-N30, and [3] P45, where P27 refers to the positive peak prior to N30, occurring between 22 and 28 ms after stimulus [40]. P45 is the positive peak following N30.

### Statistical analysis

Distributions of JND across the three measurements were not significantly different from normal distribution based on Shapiro-Wilk tests (*p *values > 0.05) and a 2 × 3 (group: stroke and control x measurement time [pretest, posttest and retention]) mixed ANOVA was performed to examine the change of JND over time with the comparison between the stroke and control groups. Pearson correlation coefficients (*r*) were computed for bivariate analysis of JND thresholds. Most variables (i.e. means and standard deviations of the instantaneous tracing errors for the five tracing task) on tracing errors were not normally distributed as indicated by Shapiro-Wilk test, even with one outlier (> 2 interquartile range [IQR]) removed across the tasks. The SEP variables N30 peak-to-peak amplitude, N30 latency, and P45 latency were not normally distributed as indicated by Shapiro-Wilk test. Therefore, to account for non-normal distribution, nonparametric Friedman tests were employed to examine changes in tracing errors and all SEP variables over the three SEP measurements for the stroke and control groups respectively. Kendall’s *w* was calculated to indicate the effect size of Friedman’s test by normalizing the chi square statistics obtained in the Friedman’s test by the number of participants (N) and degrees of freedom (i.e. the number of repeated measures – 1)[41]. Kendall’s *w* indicates the percentage of variance among the ranks explained by the repeated measures, similar to eta squared used in ANOVA designs. Spearman correlation coefficients (*r*_*s*_) were computed for bivariate analysis for tracing errors and SEP measures. Significance level was set at α = 0.05.

## Results

All participants successfully completed both the training and assessment sessions with no discomfort. S10 had VTF placed on the less-affected forearm instead of the affected arm to complete the training because he could not perceive passive wrist movement of the affect side without vision. For the same reason S10 could not complete the JND test.

### JND threshold as a marker of wrist proprioceptive acuity

Eleven of the twelve stroke participants completed the threshold testing across the three visits except S10. At pretest, mean JND was 1.8° [SD: 0.54°] for the stroke and 1.5° [0.46°] for the control group with seven stroke participants exhibiting a JND threshold higher than the median JND threshold of the control group (see Fig. 3a). At posttest, 8 out of 11 stroke participants (73 %) lowered their thresholds (see Fig. 3), which reduced the JND threshold at group level (mean: 1.4° [SD: 0.54°]). The 8 participants, who responded to somatosensory training, reduced their JND threshold between 9.3 and 47.0 % (mean: 30.2 %). Two participants showed a slight increase (2.5–6.6 %) in threshold, while one participant increased his threshold by 48 %. At retention, the reduction in JND threshold remained stable (mean: 1.3° [SD: 0.28°] for the stroke group), indicating that improvements in JND threshold persisted two days after the intervention. Mauchly’s Test of Sphericity indicated that the assumption of sphericity had been not violated (χ^2^(2) = 1.40, *p* = 0.497) and therefore no correction was used for 2 × 3 mixed ANOVA on JND. Aligned with the above observation, the analysis revealed a significant main effect of measurement time (F[2, 37] = 4.54, *p* = 0.017, η_p_^2^ = 0.19) while indicating no group and time interaction (F[2, 37] = 1.74, *p* = 0.189, η_p_^2^ = 0.08) and no main effect of group (F[1, 19] = 0.12, *p* = 0.736, η_p_^2^ = 0.06). It suggested significant reduction in JND across measurement time for both groups. Trend analysis suggested a significant linear trend for the changes across measurement time (F[1, 19] = 6.60, *p* = 0.019, η_p_^2^ = 0.26). A high pretest JND threshold was the strongest predictor of improvement in JND at posttest (*r* = − 0.71, *p* = 0.015) and at retention (*r* = − 0.80, *p* = 0.003; Fig. 3b).

**Fig. 4:**
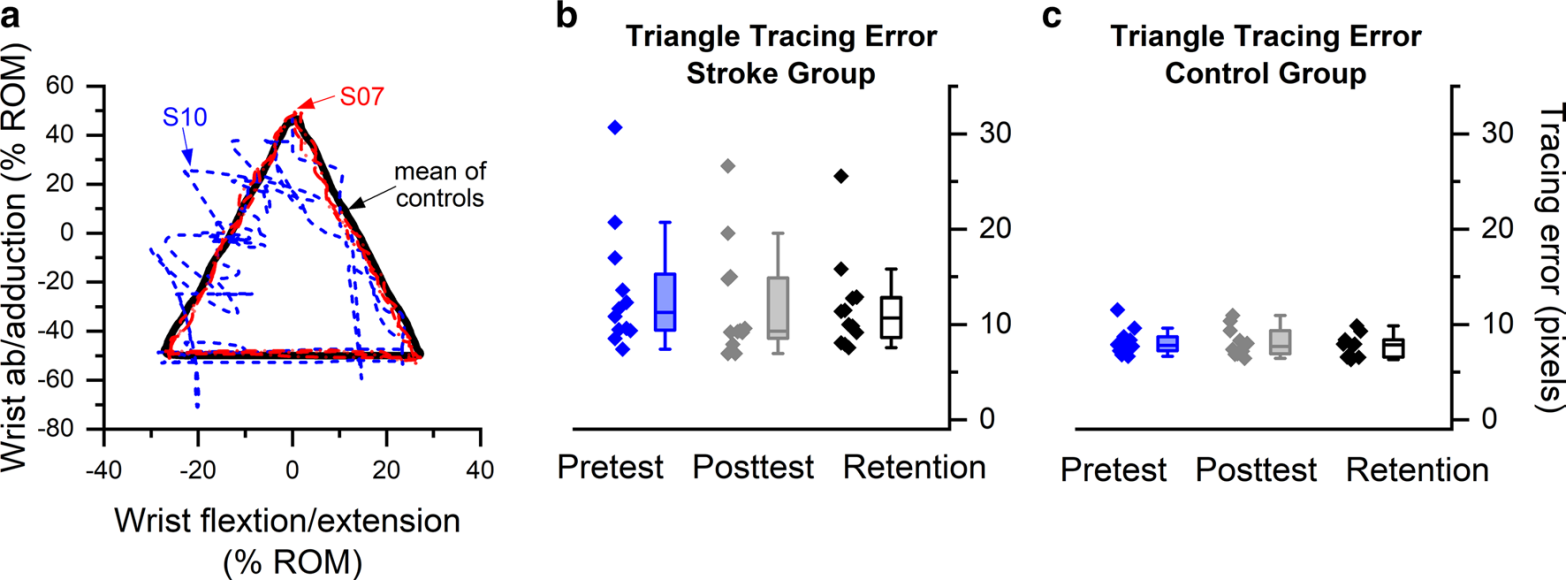
**a** Exemplar wrist tracing performance at pretest for two stroke participants (S07, S10) during the triangle tracing task. The black triangle represents the mean trajectory of the controls. S07 demonstrated comparable performance to controls as indicated in the red dashed line, while S10 exhibited a large tracing error as indicated in the blue dashed line. **b** Boxplot of triangle tracing error at pretest, posttest and retention. Each box indicates the distribution between the 25th and 75th percentiles. The line within the box indicates the median. Whiskers represent the 1st and 99th percentile. Adjacent diamond symbols show all individual subject mean tracing errors values

### Wrist tracing accuracy as a marker of untrained visuomotor performance

At pretest, the stroke group exhibited significantly higher tracing errors when tracing the triangle (*U* = 17, *p* = 0.005), figure-of-eight (*U* = 22, *p* = 0.012) and ellipse (*U* = 22, *p* = 0.012) compared to the control group. At posttest, participants of the stroke group showed 22 % reduction in tracing error on average compared to pretest. However the observed reduction did not achieve statistical significance as indicated by Friedman’s tests (χ^2^(2) = 0.30–3.17, *p *values = 0.205–0.407 for the stroke group; χ^2^(2) = 1.80–5.00, *p *values = 0.082–0.407 for the control group). Effect sizes indicated by Kendall’s *w* ranged from 0.01 to 0.13 for the stroke group and 0.09–0.25 for the non-stroke group, indicating low to medium effects. Vertical line and triangle tracing exhibited the highest effect size in the stroke group (Fig. 4b). Pretest tracing error of the ellipse correlated significantly with JND threshold at pretest (*r*_*s*_ = − 0.62, *p* = 0.043) among the stroke group, indicating association between proprioceptive acuity and motor tracing performance.

**Fig. 5:**
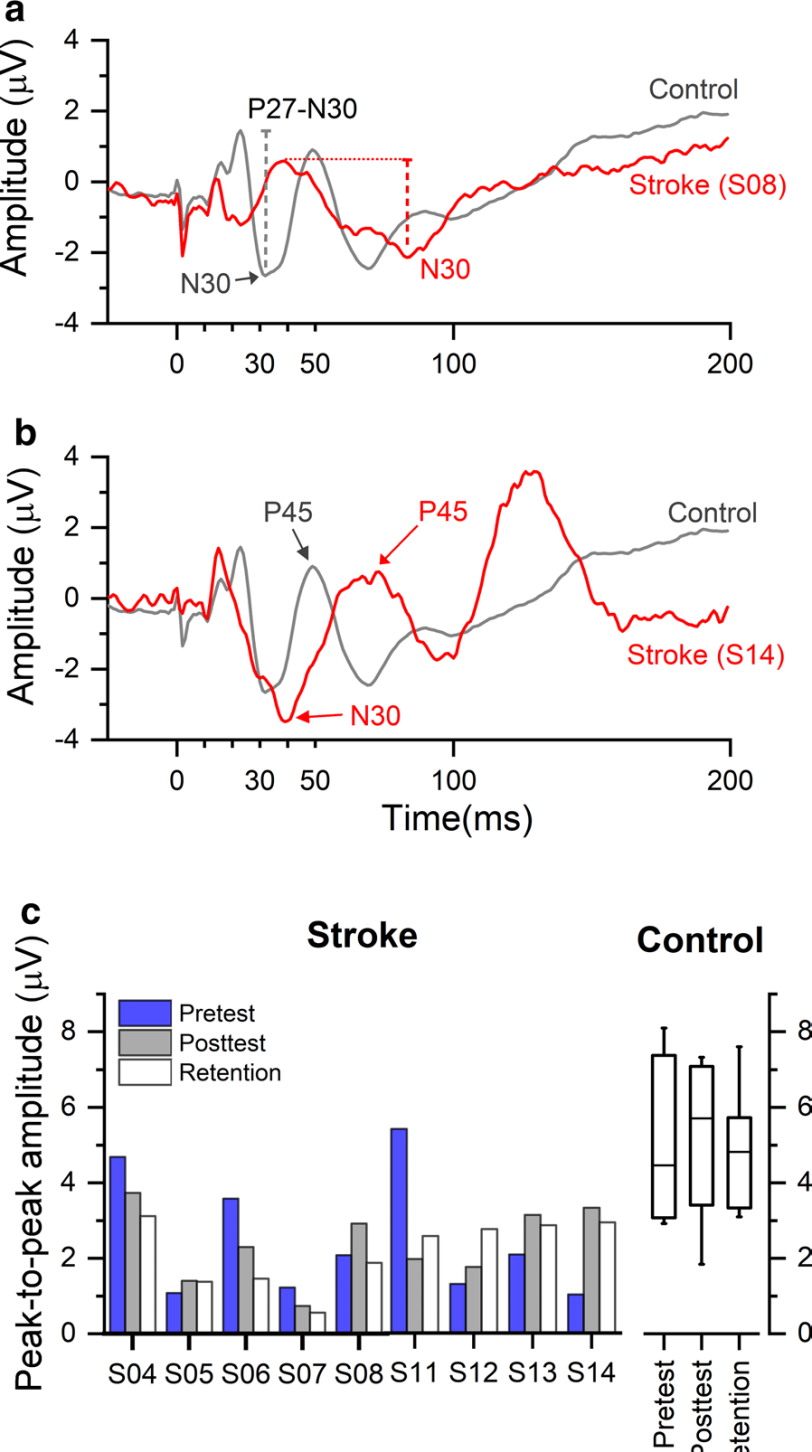
Median nerve SEP time-series data of two stroke participants and related summary data. a Stroke participant S08 exhibited a longer N30 latency and a decreased P27-N30 SEP amplitude in comparison to the average waveform of the control group. b Stroke participant S14 showed prolonged N30 and P45 latencies when compared to controls. c P27-N30 peak-to-peak amplitude across visits. Data of individual stroke participants and the summary statistics of the control group were shown: medians (lines), IQR (box boundary) and 5th−95th percentiles (whiskers)

### SEP as a marker of somatosensory processing

SEP waveforms were obtained from FC1 for optimal observation of the N30 waveform for the nine stroke participants and seven control participants who completed the SEP test. The remaining participants declined due to time pressure and fatigue as SEP was conducted last. At pretest, participants with stroke showed a significantly reduced P27-N30 peak-to-peak amplitude (median: 2.07 µV [IQR: 2.94 µV]) when compared to controls (4.47 µV [3.84 µV], *U* = 11, *p* = 0.03; Fig. 5a, c). After training, median P27-N30 peak-to-peak amplitude for the stroke group increased from 2.07 µV (2.94 µV) at pretest to 2.29 µV (1.92 µV) at posttest and to 2.87 µV (1.73 µV) at retention (40 % increase) but this increase failed to reach statistical significance at group level (χ^2^(2) = 2.00, *p* = 0.37, *w* = 0.11). In contrast of the significant group difference of P27-N30 peak-to-peak amplitude, N30 latency of six in nine stroke participants were within the range of the control group. After training, median N30 latency of both groups did not change at posttest (stroke: 39 ms [10 ms]; control: 32 ms [7 ms]) and retention (stroke: 39 ms [12 ms]; control: 32 ms [5 ms]) compared to pretest (stroke: 39 ms [9.5 ms]; control: 32 ms [7 ms]). This was confirmed by Friedman’s tests (stroke: χ^2^(2) = 0.58, *p* = 0.75, *w* = 0.03; control: χ^2^(2) = 1.08, *p* = 0.58, *w* = 0.08). Further subgroup analysis found that at pretest four of the five stroke participants with more affected proprioceptive acuity (S05, S06, S08, S14; JND threshold > = 1.8°) showed delayed latency of either N30 or P45 component. In this subgroup, a longer P45 peak latency was correlated with a higher JND threshold at pretest (*r*_*s*_ = 0.66, *p* < 0.05; Fig. 5b for example).

## Discussion

This study sought to provide initial data on the assumed effectiveness of somatosensory-focused, active movement interventions for improving sensorimotor function in chronic stroke. The main findings of the study are as follows: First, a short, 2-day movement training that challenges the proprioceptive system lead to measurable improvements in wrist position sense accuracy that persisted for at least 3 days past training. Second, evidence for a transfer of such sensory learning to the motor domain is limited. Stroke participants exhibited a 22 % mean reduction in tracing error, which is promising. However, given the high response variability of the stroke group, this difference was not statistically significant. Third, a reduced SEP amplitude during early somatosensory processing is a marker of abnormal proprioceptive processing. However, the short training did not induce a systematic change in SEP amplitude as a sign of normalized cortical processing.

### A somatosensory-focused movement training can improve proprioceptive acuity in stroke survivors

We found that 73 % (8/11) of stroke participants responded to the two-day somatosensory-based training. For those responders, wrist proprioceptive acuity improved by 30 % on average. Importantly, these gains in sensory acuity persisted for two days. This finding is encouraging as it demonstrates that a brief somatosensory-focused training can induce measurable benefits in proprioceptive function in chronic phase stroke survivors who had limited motor impairment. Our data provide initial evidence that for adults with chronic stroke an active, somatosensory-focused movement training may require less training time to achieve comparable proprioceptive improvement than either somatosensory discrimination training alone or functional arm use exercises combined somatosensory discrimination training. Research for those types of training reported that a reduction of 21–67 % in joint reposition error took between 8 and 40 h of intervention [42–45]. In comparison, the somatosensory-based movement intervention in this study yielded a comparable magnitude of proprioceptive improvement after a total of 48 min of practice time. Our approach might be more efficient for two reasons: (1) It required and relied on proprioceptive processes throughout the training time in order to perform controlled movements with vision occluded and (2) the training ask became progressively difficult and therefore continued to challenge the participants throughout the training period. On the other hand, three participants with stroke did not show improvement in the proprioceptive acuity at the posttest. They also had the most accurate proprioceptive acuity at pretest (Fig. 5c) and thus it might be possible that the intervention task did not challenge these participants sufficiently.

It is important to note that the observed proprioceptive improvement is unlikely a mere practice effect of the assessment task. While the intervention required active wrist movement, the joint position discrimination task to assess proprioceptive function is based on passive motion induced by the robot. More importantly, it is inherent to psychophysical threshold testing that the perceiver receives no feedback. That is, after two distinct joint positions were assumed during our procedure, no feedback was given about the correctness of the participant’s perceptual judgement. Thus, while one could not exclude some form of implicit learning did occur, it remains questionable what exactly was learned in a task that provided no feedback on performance or results. It is unlikely that the systematic gains in proprioceptive acuity attributes primarily to a practice effect of the assessment task.

While further studies are needed to investigate whether this approach improves proprioception and motor performance among people with limited active wrist movement after stroke, similar intervention could still be delivered by modifying the movement requirement of the training task for people who have active wrist control. More practice sessions are likely needed to achieve similar intensity of training to accommodate for the reduced movement speed. Additional assistive force shall be considered to provide actual movement assistance and augmented proprioceptive feedback. For people with no active wrist control, the proprioceptive training used in this study is not feasible and therefore alternative methods shall be sought.

### Evidence of a motor transfer

We employed a set of motor tasks that were not part of the practice regimen but used the same joint to understand if such somatosensory-focused training would transfer to untrained motor patterns. Stroke participants as a group showed on average a 22 % reduction in the tracing error at posttest when compared to the pretest. However, despite a medium level of effect size this difference was not statistically significant. There are likely two reasons that account for the absence of a significant intervention effect. First, given the large between-subject movement variability generally observed in stroke survivors, the sample size of our study was insufficient. Second, the duration of the intervention was simply too short to induce systematic improvements in untrained motor tasks. Research that employed somatosensory discrimination training combined with active somatosensory-relevant exploration activities [46] reported that 18–27 h of intervention were necessary to yield an approximately 50 % improvement in functional arm use in chronic stroke survivors with somatosensory impairment. That is, the research applying a somatosensory-focused training in a longer, more intensive regimen is necessary to generate conclusive evidence that such training can transfer to untrained functional motor tasks.

### SEPs reflect proprioceptive acuity at pretest

We recorded median nerve stimulation induced SEPs to obtain a neurophysiological signal related to cortical proprioceptive processing. We investigated if electrocortical measures derived from these SEP signals were abnormal in the stroke group and if they correspond to the psychophysical position sense thresholds that measured wrist proprioceptive acuity. Specifically, we looked at markers of early somatosensory processing. Particularly, N30 is believed to be generated in the secondary somatosensory cortex [47] and evoked by the proprioceptive input [47]. We found that the stroke group showed a significantly reduced P27-N30 peak-to-peak amplitude prior to training. In addition, in those stroke participants who were most affected proprioceptively, the P45 latency tended to be delayed—at pretest P45 peak latency strongly correlated with JND threshold. However, we found inconclusive evidence that these SEP components of early somatosensory processing were altered systematically at the end of our somatosensory-focused movement training.

## Conclusions

This study documented that proprioceptive function is trainable and can improve in chronic stroke survivors, while the effects on motor function are encouraging but inconclusive. If proven effective in future clinical trials, such intervention or its elements could be employed in clinical practice to complement existing approaches.

## Data Availability

The datasets used and/or analyzed during the current study are available from the corresponding author on reasonable request.

## Abbreviations

ADL: Activities of daily living
VTF: Vibro-tactile feedback
AA: Abduction/adduction
FF: Flexion/extension
JND: Just-noticeable difference
SEP: Somatosensory-evoked potentials
ANOVA: Analysis of variance
IQR: Interquartile range
