# Correction to: Effects of a robot‐aided somatosensory training on proprioception and motor function in stroke survivors

**DOI:** 10.1186/s12984-022-01057-9

**Published:** 2022-07-18

**Authors:** I.-Ling Yeh, Jessica Holst-Wolf, Naveen Elangovan, Anna Vera Cuppone, Kamakshi Lakshminarayan, Leonardo Cappello, Lorenzo Masia, Jürgen Konczak

**Affiliations:** 1 Health and Social Sciences Cluster, Singapore Institute of Technology, Singapore, Singapore; 2 Human Sensorimotor Control Laboratory, School of Kinesiology, University of Minnesota, Minneapolis, USA; 3 Department of Robotics, Brain and Cognitive Sciences, Istituto Italiano di Tecnologia, Genoa, Italy; 4 Department of Neurology and School of Public Health, University of Minnesota, Minneapolis, USA; 5 The BioRobotics Institute, Scuola Superiore Sant’Anna, Pisa, Italy; 6 Department of Excellence in Robotics and AI, Scuola Superiore Sant’Anna, Pisa, Italy; 7 Institut für Technische Informatik, Universität Heidelberg, Heidelberg, Germany

## Correction to: J NeuroEngineering Rehabil (2021) 18:77 https://doi.org/10.1186/s12984-021-00871-x

Following the publication of the original article [1], the author name ‘Leonardo Cappello’ has been misspelled as ‘Leonardo Capello’.

The original article has been corrected.
